# Clinico-pathological Features of Colon Cancer Patients Undergoing Emergency Surgery: A Comparison Between Elderly and Non-elderly Patients

**DOI:** 10.1515/med-2019-0082

**Published:** 2019-10-02

**Authors:** Gianluca Costa, Barbara Frezza, Pietro Fransvea, Giulia Massa, Mario Ferri, Paolo Mercantini, Genoveffa Balducci, Antonio Buondonno, Aldo Rocca, Graziano Ceccarelli

**Affiliations:** 1 Surgical and Medical Department of Translational Medicine, Sapienza University of Rome, Sant’Andrea Hospital, Via di Grottarossa 1035-39, 00189 Rome, Italy; 2 Department of Surgery, Division of General Surgery, San Donato Hospital, via Pietro Nenni 20-22, 52100 Arezzo, Italy; 3 Colorectal Surgical Oncology, Istituto Nazionale per lo Studio e la Cura dei Tumori “Fondazione Giovanni Pascale” IRCCS, Naples, Italy; 4 Department of Medicine and Health Sciences “V. Tiberio”, University of Molise, Campobasso, Italy

**Keywords:** Colorectal cancer, Emergency surgery, Elderly, Lymph node ratio, Clinicopathological features

## Abstract

**Background:**

Colorectal cancer (CRC) is one of the most common cancers in patients older than 65 years. Emergency presentation represents about 30% of cases, with increased morbidity and mortality rates. The aim of this study is to compare the perioperative outcome between elderly and non-elderly patients undergoing emergency surgery.

**Method:**

We retrospectively analysed CRC patients that underwent emergency surgery at the Departments of Surgery of the Sapienza University Sant’Andrea Hospital in Rome, and at San Donato Hospital in Arezzo, between June 2012 and June 2017. Patients were divided into two groups: non-elderly (< 65 years) and elderly (≥ 65 years). Variables analysed were sex, onset symptoms, associated disease, ASA score, tumor site and TNM stage, surgical procedures and approach, and morbidity and mortality.

**Results:**

Of a total of 123 patients, 29 patients were non-elderly and 94 patients were elderly. No significant differences were observed in sex, onset symptoms and tumor site between the two groups. Comorbidities were significantly higher in elderly patients (73.4% vs 41.4%, p<0.001). No significant differences were observed between the two groups in surgical approach and the rate of one-stage procedures. Elderly patients were more frequently treated by Hartmann’s procedure compared to non-elderly patients (20.2% vs 6.9%). Left colorectal resection with protective ileostomy was most frequent in the non-elderly group (27.6% vs 11.7%). No significant differences were found in the pT and pN categories of the TNM system between the two groups. However, a higher number of T3 in non-elderly patients was observed. A consistent number of non-oncologically adequate resections were observed in the elderly (21.3% vs 3.5%; p<0.03). The morbidity rate was significantly higher in the elderly group (31.9 % vs 3.4%, p<0.001). No significant difference was found in the mortality rate between the two groups, being 13.8% in the elderly and 6.9% in the non-elderly.

**Conclusions:**

Emergency colorectal surgery for cancer still presents significant morbidity and mortality rates, especially in elderly patients. More aggressive tumors and advanced stages were more frequent in the non-elderly group and as a matter it should be taken into account when treating such patients in the emergency setting in order to perform a radical procedure as much as possible.

## 1 Introduction

Colorectal cancer (CRC) is the second most common cancer in men and women in developed countries, in many cases arising from benign lesions due to genetic or non-genetic imbalance [1,2]. More than 70% of CRC are found in patients aged 65 years or older and the number of older CRC patients is expected to greatly increase over the coming decades [3].

Approximately one-third of CRC patients present as an emergency [4] and despite advances in surgical technology and postoperative treatment, emergency CRC resection remains a high-risk procedure, especially in elderly patients with morbidity and mortality rates of 11-35% and 9-22%, respectively.

Age, comorbidities, American Society of Anesthesiologists (ASA) score, among other factors, have been reported as significant risk factors. However, age alone predicts poor tolerance of cancer treatment and the heterogeneity of the older cancer population requires a tailored approach that considers individual frailty, especially in the emergency setting [5].

In the present study, we aimed to compare the clinical findings and perioperative outcomes after emergency surgery for CRC in the elderly population with those observed in younger patients.

## 2 Patients and methods

We retrieved data from the Operating Room Registry of the Department of Surgery of San Donato Hospital in Arezzo and from the Acute Care Surgery Registry of the University Hospital Sant’Andrea of Rome[6]. For the purpose of this study, patients undergoing emergency surgery for colorectal cancer from June 2012 to June 2017 were analyzed. Colorectal procedures were selected on the basis of the International Classification of Diseases (ICD-9-CM) [codes 45.4x, 45.52, 45.7x, 45.8, 46.94, 48.62, 48.63]. Emergency resections were defined on the basis of the National Confidential Enquiry into Patient Outcome and Death classification (NCEPOD) [7]. In an attempt to avoid bias in the research, only the patients treated in a dedicated operating room within 48 hours of admission were considered eligible for this study. The above time limit was determined following the suggestion of the Timing of Acute Care Surgery classification proposed by the World Society of Emergency Surgery [8]. Patients were initially split into two main groups, named elderly and non-elderly group. The elderly group was comprised of patients of 65 years of age or above, while the non-elderly group was comprised of patients under 65 years. The elderly group patients were in turn divided into three subgroups based on age as follows: Subgroup A: 65-74ys, Subgroup B: 75-84ys and Subgroup C: >85ys. All patient records were reviewed, including demographics (sex and age at surgery), clinical data (ASA score, BMI), onset of symptoms, comorbidities, surgical procedures, tumor location and pathological features. Onset symptoms were defined as follows: obstruction, acute abdominal symptoms (encompassing peritonitis, abscess and/or overt perforation), and bleeding. All patients were histopathologically diagnosed with primary adenocarcinoma. Tumour site was defined as right or left using the middle colic artery as landmark. Tumors located below the peritoneal reflection were excluded. Pathological data included the T and N stage of colorectal cancer, grading, the lymph node harvest (LNH) and the lymph node ratio (LRN) according to well described criteria [9]. A formal Institutional Review Board approval was not required because of the non-interventional retrospective design; however, a consent for the collection of data for scientific purpose was obtained from all patients at hospital admission.

### 2.1 Surgery

All procedures were performed by senior staff surgeons following the same oncologic principles, such as main vascular pedicle ligation to achieve the best nodal clearance with adequate bowel margins and whenever possible the maximum same extent of resections. The vessels were dissected and ligated up to the origin of the superior or inferior mesenteric pedicle [6].

Antibiotics and hydration fluids were administered according to patients’ clinical conditions. The antibiotic cefazolin (plus metronidazole in left sided resections) was always administered starting at admission; the antibiotic was then suspended after the third postoperative day, if there were no signs of infection. Patients were allowed to consume liquids when their bowel sounds became evident, and they were progressed to a solid diet as tolerated. Patients were discharged after the passage of the first stool, when fully ambulatory and tolerant of a solid diet.

### 2.2 Outcome measures

The primary outcome measures were 30-day morbidity and mortality rates. The secondary outcome measures included the oncologic adequateness of the resection, and the length of hospital stay. The surgical procedure was considered as curative if R0 resection was detected at the histopathological examination and if the number of lymph nodes harvested was >12. Hence, we considered patients who underwent no tumour removal, doubtful or grossly inadequate local clearance, and patients who were found to have microscopic residual disease (R1) or LNH < 12 to have had a non-curative treatment. Due to the wide variability of the situations defining the treatment as non-curative, we preferred to classify the resection as Curative (C-Res) or Non Adequate (No Adeq-Res) (Table 1). The Clavien-Dindo classification system was used to categorize complications.

**Table 1:** Patients’ demographics and clinical data.

	Non-Elderly (29 patients)	Elderly (94 patients)	P	65-74 yrs 47 (50%)	75-84 34 (36.2%)	>85 13 (13.8%)	P
Gender			ns	24 (51.0%)	16 (47.0%)	5 (38.4(%)	
Male	14 (48.3%)	45 (47.9%)		23 (49.0%)	18 (53.0%)	8 (61.5%)	0.718
Female	15 (51.7%)	49 (52.1%)					

Onset symptoms			Obstr. vs. Perf.				
Obstruction	26 (89.7%)	71 (75.5%)		35 (74.4%)	28 (82.3%)	8 (61.5%)	0.133
Perforation	1 (3.4%)	17 (18.1%)	=0.068	8 (17.0%)	4 (11.7%)	5 (38.4%)	
Bleeding	2 (6.9%)	6 (6.4%)		4 (8.51%)	2 (5.88%)	0 (0%)	

ASA score			<0.001				
1	5 (17.2%)	5 (5.3%)		4 (8.51%) 19	1 (3.0%)	0 (0%)	<0.001
2	17 (58.6%)	25 (26.6%)		(40.4%) 23	6 (17.6%)	0 (0%)	
3	7 (24.1%)	50 (53.2%)		(49.0%)	22 (64.7%)	5 (38.4%)	
4	0 (0.0%)	14 (14.9%)		1 (2.1%)	5 (14.7%)	8 (61.5%)	

Associated disease	12 (41.4%)	69 (73.4%)	<0.002	25 (53.2%)	31 (91.2%)	13 (100%)	<0.001
Cardiovascular disease	10 (34.5%)	53 (56.4%)		18 (38.3%)	24 (70.6%)	11 (84.6%)	
Respiratory disease	6 (20.6%)	28 (29.9%)		13 (27.6%)	8 (23.5%)	7 (53.8%)	
Diabetes	4 (14.0%)	25 (26.6%)		8 (17.0%)	12 (35.3%)	5 (38.5%)	
Chronic renal failure	2 (6.9%)	9 (9.6%)		1 (2.12%)	2 (5.9%)	6 (46.1%)	
Obesity (BMI>30)	5 (17.2%)	9 (9.6%)		5 (10.6%)	4 (11.7%)	5 (38.4%)	
History of Tumour	1 (3.5%)	6 (6.4%)		1 (2.1%)	3 (8.8%)	2 (15.4%)	
Immunodeficiency	0 (0.0%)	3 (3.2%)		2 (4.2%)	1 (2.9%)	0 (0%)	
Other	0 (0.0%)	5 (5.3%)		2 (4.2%)	1 (2.9%)	2 (15.4%)	

Tumour site			ns	16 34.4%)	12 (35.3%)	4 (30.8%)	ns
Right colon	12 (41.4%)	32 (34.0%)		31 (91.2%)	22 (64.7%)	9 (69.2%)	
Left colon	17 (58.6%)	62 (66.0%)					

T stage			ns				ns
Tis	0 (0.0%)	1 (1.1%)		1 (2.1%)	1 (3.0%)	0 (0%)	
T1	0 (0.0%)	2 (2.1%)		1 (2.1%)	1 (3.0%)	0 (0%)	
T2	0 (0.0%)	3 (3.2%)		1 (2.1%)	2 (5.8%)	0 (0%)	
T3	22 (75.8%)	44 (46.8%)		21 (44.7%)	15 (44.1%)	8 (61.5%)	
T4	7 (24.1%)	44 (46.8%)		26 (55.3%)	19 (55.9%)	3 (23.1%)	

N stage			P=0065				<0.001
N0	8 (27.7%)	37 (42.5%)		27 (57.4%)	10 (29.4%)	0 (0%)	
N1	8 (27.7%)	32 (36.8%)		18 (38.3%)	10 (29.4%)	4 (30.7%)	
N2	12 (41.6%)	18 (20.7%)		2 (2.1%)	14 (41.2%)	9 (69.2%)	

Lymph node harvest (LNH)	21.64 ± 5.41	17.34 ± 9.46	P<0.03	17.94 ±11.28	17.31 ±10.39	11.23 ±9.60	ns
Lymph node ratio (LNR)	0.165 ± 0.219	0.155 ± 0.234	P=ns				
LNH >12	28 / 28 (100.0%)	75 / 89 (84.3%)	P<0.03				

Oncologically adequacy of							
surgery			P<0.03	39 (83.0%)	30 (88.2%)	5 (38.5%)	<0.001
Curative (C-res)	28 (96.5%)	74 (78.7%)		8 (17.0%)	4 (11.8%)	8 (61.5%)	
Non Adequate (NoAdeq-res)	1 (3.5%)	20 (21.3%)					

Surgical approach			P=ns				ns
Open	22 (75.9%)	80 (85.1%)		38 (80.8%)	33 (97.0%)	9 (69.2%)	
Laparoscopic	7 (24.1%)	11 (11.7%)		7 (15.0%)	1 (3.0%)	3 (23.1%)	
Laparoscopic converted	0 (0.0%)	3 (3.2%)		2 (2.1%)	0 (0%)	1 (7.7%)	

### 2.3 Statistical analysis

Statistical analysis was carried out using the 21.0 version of the IBM-SPSS Statistics Programme (IBM Analytics Italy, Segrate, MI) for MacOsX and using statistical calculators from www.socscistatistics.com Continuous data are presented as mean values plus standard deviations. Dichotomous data and counts are presented in frequencies and percentages. On univariate analysis, elderly and non-elderly patients were compared for the clinical variables and outcome measures using the Fisher exact test, the chi-square test with or without Yates’s correction, and the Mann-Whitney U test when appropriate. Furthermore, the elderly subgroups of patients were compared and then each subgroup was compared with the group of non-elderly patients by using the chi-square calculator for a contingency table and the ANOVA test when appropriate. The tests performed were two-tailed and a p value <0.05 was considered as statistically significant.

### 2.4 Results

In the study period, a total of 123 patients undergoing emergency surgery for colorectal cancer were considered for analysis because they satisfied the inclusion criteria. Of those, 64 were female (52.0%) and 59 were male (48.0%). The mean age was 72.3 ± 13.2 years (range 37-94 years). The non-elderly group comprised 29 (23.5%) patients, while 94 (76.5%) patients were assigned to the elderly group. Patients’ demographics and clinical data are summarized in Table 1.

No difference was observed between the two groups with regard to sex. The most frequent onset symptom was obstruction in both groups but a considerably relative high rate of perforation in the elderly group (18.1% vs 3.4%) had been observed, even if the difference did not reach statistically significance.

As expected, elderly patients were more often affected by comorbidities (73.4% vs 41.4%, p<0.002), especially cardiovascular disease (56.4%) and as a consequence, elderly patients were significantly more frequently classified as ASA III (53.2%) compared with non-elderly patients (24.1%). It is relevant to consider the morbid obesity rate in the non-elderly group. No significant differences were found in tumour location between the two groups, but in the elderly population tumours were predominantly left sided (66.0% vs 58.6%). The overall rate of advanced stage (T3-T4) was 95.2%. Although the incidence of T3 stage in the non-elderly group was higher than in elderly population (75.9% vs 46.8%) the difference did not reach statistical significance.

No significant difference was observed between the two groups in pN category of the TNM staging system. It was noteworthy that most patients in the non-elderly group presented as pN2 category compared to the elderly group (41.6% vs 20.7%). The mean lymph node harvest (LNH) number was significantly higher in the non-elderly group, compared to the elderly group (21.64±5.41 vs 17.34±9.46, respectively; p<0.03). There was no statistically significant difference in Lymph Node Ratio (LNR) between the two groups. When considering patients submitted to resection and suitable for LNH adequacy, all patients in the non-elderly group were found to have more than 12 lymph node retrieved, while only 75 patients (84.3%) in the elderly group were found to have had the same. The difference was statistically significant (p<0.03). No statistically significant difference emerged from the comparison between the non-elderly group with the subgroup A of elderly patients. The difference becomes slightly but not significantly significant with the subgroup B (p=0.66), reaching a statistical significance when comparing the group of non-elderly with the subgroup C (p<0.03). One patient in the elderly group was found to have microscopic residual disease (R1), while all patients in the non-elderly group underwent R0 resection. Surgical procedures did not significantly differ between the elderly and non-elderly patients. The list of procedures performed is shown in Table 2. A similar rate of right hemicolectomy and left colorectal resection with primary anastomosis was encountered in both groups. Hartmann’s procedure was more frequently performed in the elderly group (20.2% vs 6.9%), while left colorectal resection with protective ileostomy was the most frequent procedure done in the non-elderly group (27.6% vs 11.7%). There were no differences in surgical approach (open procedure vs laparoscopic resec

**Table 2:** Procedure list.

	Non Elderly	Elderly	P	65-74 47 (50%)	75-84 34 (36.2%)	>85 13 (13.8%)	P
Right hemicolectomy	11 (37.9%)	31 (33.0%)	ns	16 (34.0%)	12 (35.3%)	3 (23.1%)	ns
Left resection and primary anastomosis	7 (24.1%)	24 (25.5%)	ns	17 (36.2%)	6 (17.6%)	1 (7.7%)	<0.05
Left resection and primary anastomosis with protective ileostomy	8 (27.6%)	11 (11.7%)	ns	6 (12.8%)	5 (14.7%)	0 (0%)	ns
Hartmann’s procedure	2 (6.9%)	19 (20.2%)	ns	3 (6.4%)	9 (23.5%)	7 (53.8%)	<0.001
Stoma only	1 (3.4%)	5 (5.3%)	ns	1 (2.1%)	2 (5.8%)	2 (15.4%)	ns
Others	0 (0.0%)	4 (4.3%)	ns	4 (8.5%)	0 (0%)	0 (0.0%)	ns

tion) between the two groups. Open procedures were per- 22 patients (75.9%) in the non-elderly group. In the elderly formed in 80 patients (85.1%) in the elderly group and in group, a non-oncologically adequate resection was achieved more frequently than in the non-elderly group (21.3% vs 3.5%, p<0.03).

When considering the surgical treatment performed, a statistically significant higher number of Hartmann procedures were done in the very elderly patients (subgroup D) while a significantly higher number of left resections with primary anastomosis were performed in the elderly patient subgroups A and B. The overall mean LOS was 14.7 ± 11.0 (range 3-73 days). The mean LOS was 10.2 ± 3.2 (range 6-17 days) in the non-elderly group and 15.7 ± 11.8 (range 3-73 days) in the elderly group. The difference was statistically significant (p<0.05). The overall morbidity and mortality rates were 25.2% (31 patients) and 12.2% (15 patients) in the elderly and non-elderly group, respectively. The morbidity rate was significantly higher in elderly patients compred to the non-elderly group (31.9 % vs 3.4%, p<0.001). Mortality rates in the elderly and non-elderly groups were 13.8% (13 patients) and 6.9% (2 patients) respectively (p=0.3641; Odds Ratio 2.167; 95% CI=0.459-10.219).

**Ethics approval and consent to participate**: All of the procedures in studies involving human participants were performed in accordance with the ethical standards of the institution and were in accordance with the 1964 Declaration of Helsinki and its later amendments or comparable ethical standards. Informed consent for the surgical procedures was obtained before treatment from each of the participants who were included in the study.

**Consent for publication**: Consent was provided by each of the participants enrolled in our study.

## 3 Discussion

The elderly population is steeply increasing worldwide and it presents notable challenges when planning and delivering healthcare services. There is no agreement on a definition of elderly. In the literature the cut-off for a definition of elderly varies from being either 65 or 75 years of age. Many studies define as “elderly” only patients older than 80 years [10, 11, 12, 13, 14, 15]. At present, the WHO still consider as elderly those individuals of 60 years or over; however most scientific societies define patients as elderly if their age is 65 year or more. After a literature review, we have chosen 65 years as an initial cut-off to define patients as elderly according to the decision by other Italian scientific societies (16, 17).

Elderly patients have an increased operative risk and high postoperative morbidity and mortality rate because of associated diseases such as hepatic, cardiovascular, and pulmonary diseases [18, 19, 20, 21, 22]. These patients are often also called “frail”. Frailty is defined as a condition of vulnerability strictly associated with an increased risk of poor outcomes [23, 24, 25]. In the literature, the “frailty phenotype” is a condition defined by the presence of five criteria. These are: unintentional weight loss, self-reported exhaustion, weakness (grip strength), slow walking speed and low physical activity.

A lot of pathogenic mechanisms could be proposed to explain frail phenotype onset. One of most promising is the elevation of ROS (Reactive oxygen species) and oxidative stress [26,27]. These factors are increased in several age-related diseases [28, 29, 30]

In the emergency setting, the frailty of the elderly becomes more critical and any emergency surgery has to be considered a very high risk procedure because it may suddenly put out of balance an otherwise normal subject.

Therefore, when assessing risk factors for surgery, it would be better to consider biological age rather than chronological age [29]. Many studies have demonstrated that age alone is not a prognostic factor in short-term outcomes and survival after colonic surgery [31, 32, 33]. On the contrary, important factors affecting morbidity and mortality rates are emergency surgery and comorbidities. Smothers et al. stated that emergency surgery adversely affects immediate surgical morbidity and mortality, without distinguishing patients by age [34].

The significantly higher number of comorbidities is the most important predictive factor of postoperative complications or early mortality. In agreement with literature and as expected, in this study elderly patients present more comorbidities than younger ones and they were classified as ASA III. In our experience, individual preoperative risk was assessed using ASA score because it can be quickly determined at admission and it is predictive of morbidity and mortality [21, 35]. Not unlike with other studies, the most frequent onset symptom was obstruction in both study groups. This symptom was generally associated with left sided tumours. In our experience, left sided tumours were slightly more frequent in elderly patients, but we did not find any significant differences in terms of tumour location between the two groups.

Emergency surgical treatment of colorectal cancer depends on tumour site, intraoperative findings, patient’s general condition and surgeon experience [36, 37]. With regards to approach, according to previous reports, our series shows a significantly lower number of laparoscopic approachs in elderly patients than in younger ones. Typically, patients with right-sided lesions underwent right hemicolectomy with primary anastomosis. In cases of left-sided tumours, the optimal surgical treatment is still a matter of debate and mainly includes colorectal resection and primary anastomosis, resection and anastomosis with temporary diverting ileostomy and Hartmann’s procedure. The latter one was previously considered a first-choice procedure in emergency colon surgery since it is fast to perform and prevents anastomotic leakage, but it requires a second stage to reverse the colostomy [38]. Therefore, there has been reported a shift regarding the surgical approach with a higher rate of one-stage procedure being performed [6,39, 40, 41]. Many studies, however, still report an increasing number of surgical procedures involving the creation of a diverting stoma in elderly patients [42]. Formisano et al. suggest that primary resection and anastomosis is optimal in selected patients with low risk; temporary ileostomy is indicated in patients with intermediate risk, advanced obstruction, perforation or locally advanced condition; Hartmann’s procedures should be preferred as the safer surgical procedure when doubt exists [43]. In our series no differences were found in the number of right hemicolectomy and left colorectal resections and primary anastomosis performed between the two groups. In the face of left-sided tumour and consistent risk of anastomotic leakage, Hartmann’s procedure was the preferred procedure in elderly patients while primary resection and anastomosis with diverting temporary ileostomy was the more common treatment of choice in the non-elderly group.

Colorectal tumours submitted to emergency surgery are at a more advanced stage and this finding is related to an increase in perioperative mortality rate and poor survival [44, 45]. Similarly, we report a high rate of T3 and T4 tumour stages in both groups. No statistically significant differences in N stage were observed in our study; however we reported a higher number of N+ stage, mainly N2, in the non-elderly group.

A minimum examination of 12 harvested lymph nodes is recommended to achieve correct staging [46, 47, 48]. Many studies showed that emergency surgery does not influence the adequacy of lymph node harvest [49, 50, 51], while age is a statistically significant variable affecting LNH [52, 53]. Our study shows a similar decreasing rate of adequate lymph node harvest in elderly patients; however no difference was found regarding LNR. We documented that a palliative, or better defined, non-adequate resection has been frequently performed in the elderly group, especially in the very elderly patients. We can not clearly explain this finding. Knowing that all the staff surgeons involved have colorectal surgical expertise and follow the same oncologic principles, we postulate that some surgeons’ choices could be guided by making intraoperative risk-benefit analysis leading to a less extensive procedure in the elderly patients such as the minimum bowel length resection required to reach the abdominal wall while performing Hartmann’s operation.

## 4 Conclusions

Emergency presentation of colorectal cancer is common and emergency surgery represents a high risk procedure, especially in elderly patients. The non-elderly group presented at more advanced tumor stages, but even so, they more often underwent curative resections, compared to the elderly group. Age alone should not be considered to be more of a contraindication or a worse predictor than other factors for the outcome after colorectal surgery on elderly patients. A personalized strategy is required, considering each patient’s comorbidities, performance status, and life style. Despite the recent changes in surgical approach, Hartmann’s procedure still remains an option in very elderly patients.

## Abbreviations

CRC: Colorectal cancer
ASA: American Society of Anesthesiologists
LNH: Lymph node harvest
LNR: Lymph node ratio
NCEPOD: National Confidential Enquire into Patient Outcome and Death
WHO: World health organization.
